# Carotid Blood Flow as a Surrogate for Pulse Contour Analysis in Assessment of Fluid Responsiveness: A Prospective, Observational, Single-Centre Study (Contour Study)

**DOI:** 10.7759/cureus.53253

**Published:** 2024-01-30

**Authors:** Anirban Karmarkar, Divya Pal, Deepak Govil, Sweta J Patel, Jagadeesh KN, Rahul Harne, Anant Vikram Pachisia, Pooja Tyagi, Devireddy Madhav Reddy, Keerti Brar

**Affiliations:** 1 Critical Care Medicine, Medica Superspeciality Hospital, Kolkata, IND; 2 Anaesthesiology and Critical Care Medicine, Medanta-The Medicity, Gurugram, IND; 3 Critical Care Medicine, Medanta-The Medicity, Gurugram, IND

**Keywords:** passive leg raise, flotrac-vigileo, abdominal surgery, fluid responsiveness, cardiac output, pulse contour analysis, carotid blood flow

## Abstract

Background and objectives

The quest for an accurate and reliable non-invasive method of assessing cardiac output in critically ill patients is still ongoing. Carotid artery Doppler is a promising non-invasive, reproducible, and feasible bedside monitor. So we compared the change in cardiac output derived from arterial pressure waveforms (pulse contour analysis) with that from carotid artery Doppler-derived measurements, in post-major elective abdominal surgery patients.

Materials and methods

We conducted a prospective observational study in 30 adult post-major elective abdominal surgery patients admitted to the Gastroenterology and Liver Transplant intensive care unit postoperatively on mechanical ventilator support, who were found to be fluid responsive clinically on passive leg raise (PLR) test. Demographics and vasopressor support were recorded. Hemodynamic parameters including heart rate, systolic blood pressure (SBP), diastolic blood pressure (DBP), cardiac output (CO) using arterial pulse contour analysis (Vigileo monitor/FloTrac® sensor; Edwards Lifesciences, Irvine, California, United States), and carotid blood flow (CBF) were recorded on the baseline, pre- and post- PLR, and post fluid bolus administration. Balanced salt solution at the rate of 6ml/kg over 20 minutes was given as a fluid bolus.

Results

Of the 30 patients who were included in the study, 16 patients (53.3%) were on vasopressor support, mean (± SD) age of the patients was 52.93 (± 8.13) years. There was a significant increase in the SBP (mmHg) pre- to post-PLR, that is, 112.2±15.57 and 118.7±14.96, respectively (p-value = 0.001). Also from pre-PLR to post-fluid bolus administration, the increase in SBP was significant, 112.2±15.57 and 121.93±13.96, respectively (p-value = 0.001). The change in cardiac output measured using Vigileo and CBF from pre- to post-PLR (7.66±1.45 to 9.14±1.76, p< 0.001 for Vigileo and 8.10±1.66 to 9.72±1.99, p<0.001 for CBF) and pre-PLR to post fluid administration (7.66±1.45 to 9.39±1.77, p< 0.001 for Vigileo and 8.10±1.66 to 10.31±2.26, p< 0.001 for CBF) were significant. There was a positive correlation between the change in cardiac output as measured from arterial pulse contour analysis technique (Vigileo) and that measured from CBF (r=0.884) pre- and post-PLR. There was a significant correlation between cardiac output measurements derived from two techniques, before PLR, after PLR, and after fluid expansion (p< 0.001 for each variable). The change in cardiac output before PLR and after fluid expansion was also correlated by both the techniques (correlation coefficient being, r=0.781).

Conclusion

There was a significant positive correlation of the CO (absolute and change) measurements pre- and post-interventions (that is, PLR and fluid bolus administration) as made by pulse contour analysis (Vigileo) and by CBF in post-surgical patients. Pulse wave Doppler of CBF could be used as a surrogate for invasive measures of CO measurement for prediction of fluid responsiveness in this subgroup. Further larger studies can be performed to validate the same.

## Introduction

Fluid resuscitation plays a pivotal role in the management of critically ill patients but almost half of these might not be fluid-responsive [1,2]. Intravenous fluid administration should be considered just like any other pharmacological agent administration, and the concept of four Ds, that is drug, dose, duration, and de-escalation should be followed. To avoid loading the patients with excess fluids and consequent adverse effects, it is imperative to differentiate the fluid responders from the non-responders. For the patients who are on the flat portion of the Frank-Starling curve, overzealous fluid resuscitation wouldn’t improve the hemodynamic status, rather it may prove detrimental [3-5].

Fluid responsiveness is defined as an increase in cardiac output (CO) by 10-15% in response to fluid administration [6]. The static measures of preload such as central venous pressure haven’t shown consistency in assessing fluid responsiveness and dynamic indices have been proven to be more reliable in predicting fluid responsiveness than the static ones [7-9].

Passive leg raising (PLR) leading to an increase in CO suggests that the patient is fluid-responsive. It is carried out with the patient in a semi-recumbent position, followed by passively raising the legs at 30-45° and finally returning the patient back to the semi-recumbent position, and measurement is recorded at each step [10]. PLR leads to an auto-bolus of fluid measuring around 300 ml [11]. Since there is no actual fluid administration, the hemodynamic effects are rapidly reversible. However, it requires a real-time assessment of hemodynamic indices and CO, which is difficult to perform at bedside many a times and is invasive. Pulmonary artery catheter has traditionally been used as a reference for CO measurement [8,12] but is invasive and the associated complications may outweigh the benefits offered [13]. Minimally invasive techniques such as esophageal Doppler and pulse contour waveform analysis have demonstrated good accuracy regarding the prediction of fluid responsiveness. The non-invasive devices such as bioreactance have mixed evidence in their favor [14-16]. Bedside noninvasive measures such as echocardiographic measurement of stroke volume (SV) and CO showed good agreement with bolus thermodilution in a meta-analysis [17]. Point-of-care ultrasound (POCUS) is nowadays routinely performed in the ICUs and any clinician practising the same is expected to be able to demonstrate left ventricle outflow tract (LVOT) velocity time integral (VTI) [18]. However, in a significant number of patients, it is difficult to attain a good echocardiography window. Carotid artery Doppler, apart from being a non-invasive, reproducible, and feasible bedside monitor, provides a reasonable alternative for the assessment of fluid responsiveness by evaluating changes in carotid flow time and carotid blood flow (CBF) during inspiration and expiration. A positive correlation was found by Marik et al. between the percent change in the stroke volume index and the concomitant percent change in CBF after PLR in patients with septic shock (r=0.59; P=.0003)[19]. Ma et al. in 2017 compared two non-invasive measures (corrected carotid flow time and CBF) and their correlations with invasive reference measurements of cardiac output obtained by the thermodilution method through right heart catheterization. They found that CBF measurements correlated moderately with CO and were less influenced by measurement issues than corrected carotid flow time [20].

Gassner et al. conducted a feasibility study comparing common carotid ultrasound study for CO measurements compared to pulmonary artery catheter or pulse contour analysis in ICU patients. They concluded that using carotid Doppler can help determine CO in patients with no apparent change in cerebral circulation limiting the need for invasive monitoring for the purpose of cardiac output estimation [21]. Peng et al., in their study comparing the effectiveness of common carotid artery (CCA) sonography with transthoracic echocardiography (TTE) for CO measurements, found an overall intraclass correlation coefficient of 0.537 between the CO measurements by the two methods. But the intra-class correlation coefficients between the two were 0.241, 0.061, and 0.095, for septic shock, multiple trauma, and respiratory failure subgroups, respectively. So they concluded that CO derived from carotid artery Doppler can be used as an alternative method when it is difficult to obtain TTE images and in emergencies but they recommended against its use in above specified subgroups [22]. In a study by Roy et al., a significant decrease in carotid peak systolic velocity (CPSV) variation after fluid loading was found in fluid responders, and that corresponded with the decrease in stroke volume variation post-fluid bolus in fluid-responsive patients [23].

There are conflicting reports regarding the validity of carotid artery Doppler-derived CO measurements. In our study, we hypothesized that changes in CO from pulse contour analysis with FloTrac® sensor/Vigileo monitor (Edwards Lifesciences, Irvine, California, United States) would correlate with the changes in CO derived from CBF in patients who underwent elective major abdominal surgery.

## Materials and methods

This observational study was conducted on post-operative patients who underwent major elective abdominal surgery and were admitted to the Gastroenterology and Liver Transplant ICU at Medanta-The Medicity, Gurugram, India, between March 2020 to February 2021. The study was approved by the Medanta Institutional Ethics Committee (approval number: MICR 1038/2020 (DNB))

Sample size

For the calculation of sample size, the minimum value of the correlation coefficient was assumed as 0.5 for clinical significance.

The formula used was N= [(Zα + Zβ)/C]2 + 3, where C = 0.5 ln[(1+r)/(1-r)]

where N =sample size, r = correlation coefficient, Zα = Value of standard normal variate corresponding to an alpha level of significance, Zβ= the standard normal deviate for the desired power. With confidence level = 95% and power = 80%, the minimum sample size was calculated as 30.

Study population

Inclusion Criteria

Patients aged >18 years, admitted to ICU after elective major abdominal surgery on mechanical ventilator support, with an arterial line and Flotrac/Vigileo attached when the attending physician wants to assess fluid responsiveness by PLR test, in the presence of (i) systolic blood pressure (SBP)< 90 mmHg, diastolic blood pressure (DBP)< 60 mmHg, or mean arterial pressure (MAP)< 65 mmHg in a previously normotensive patient with vasopressors, (ii) SBP < 20% of baseline BP in hypertensive patient, (iii) heart rate >110/minute, and (iv) urine output< 0.5 ml/kg/hour, and If there is a rise in CO by more than 10% in PLR test, and PLR test was positive, were included.

Exclusion Criteria

Patients in whom PLR test could not be performed (raised intracranial pressure), or would be unreliable (intra-abdominal hypertension), who had BMI< 18.5 kg/m^2 ^or >29.9 kg/m^2^, carotid artery stenosis, aortic valvular abnormality, or chronic kidney disease on maintenance hemodialysis were excluded.

Study objective

The primary objective of the study was to study the correlation between the change in CO as measured from CBF with that from pulse contour analysis for fluid responsiveness. The secondary objective was to study the correlation of CO derived from CBF with CO derived from pulse contour analysis

Data collection

**Figure 1:**
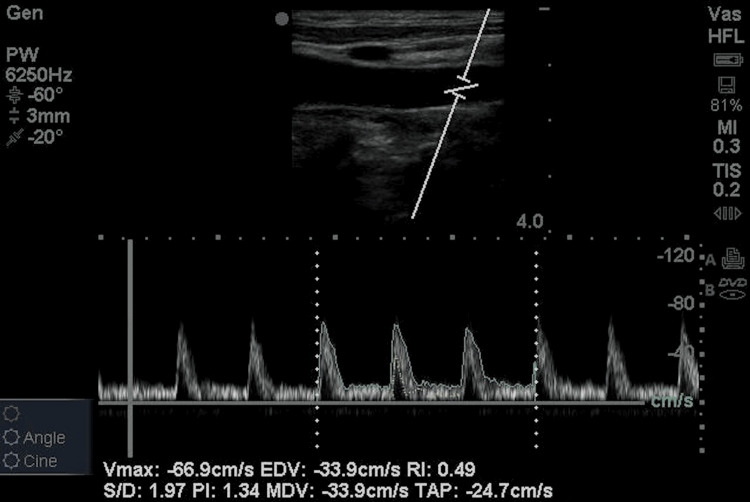
Common carotid artery spectral Doppler The  green  tracing  represents the TAMAX, also known as TAP; while the yellow tracing represents the TAMEAN; TAMAX being more than TAMEAN TAMAX: time-averaged maximum velocity; TAP: time-averaged peak velocity; TAMEAN: time-averaged mean velocity; RI: resistive index; PI: pulsatility index; EDV: end diastolic velocity; MDV: mean diastolic velocity; PW: pulsed-wave Doppler

Measurements were conducted with two physicians per patient; one physician recorded the CO from the Flotrac/Vigileo and the other performed the carotid artery Doppler; the measurements obtained from either method were blinded from each other. We used the Sonosite Edge ultrasound machine (FUJIFILM Sonosite, Bothell, Washington, United States) with a linear transducer (frequency 10 MHz) for CCA image acquisition, and images were obtained 2-3 cm proximal to the carotid bulb. The intraluminal CCA diameter was measured in a longitudinal view. Spectral Doppler tracings were then obtained by placing a 1 mm sample gate through the center of the vessel, in accordance with standard guidelines. The angle correction cursor was placed parallel to the direction of blood flow. Images with insonation angles >60° were excluded because of resultant inaccuracies of flow and velocity measurements at such angles. Carotid Doppler waveforms and the time-averaged peak velocity of three cardiac cycles were obtained with a 1 mm caliper placed parallel to the vessel walls in the center of the laminar flow identified by color flow sonography. The average of the three CBFs was calculated (Figure 1).

Volume flow = Cross-sectional area (A) × Time-averaged peak (TAP). Here, A = 𝜋𝑑2/4 , where d is the diameter of the vessel measured in a cross-sectional view. TAP velocity was calculated by drawing the cursor over the waveform, including both the systole and the diastole. CO was calculated as: CO = CBF × heart rate × 10.

PLR was performed as per the standard method and CO was noted from the Flotrac/Vigileo when it reached its maximum value/after one minute. If there was a 10% or more rise in CO, carotid artery flow measurements were taken and readings noted. Patients were once again restored to semi-recumbent and CO was noted. If the CO returned to baseline, the PLR test was considered to be positive, and patients were included in the study. The fluid bolus of balanced salt solutions at 6 ml per kg was infused over 20 minutes. CBF and CO measurements were repeated. Hemodynamic variables such as heart rate and blood pressure were recorded before PLR, after PLR, and post-fluid bolus were also recorded. Patients’ data such as age, sex, weight, height, BMI, heart rate, blood pressure, urine output in the past one hour, CO data, average CBF and CBF derived CO, and requirements for vasopressors or mechanical ventilatory support were also recorded.

Statistical analysis

Descriptive analysis of quantitative parameters was expressed as means and standard deviation (SD). Categorical data were expressed as absolute number and percentage. We used a paired student t-test for testing of mean change of paired observation. Pearson's correlation coefficient was calculated to assess the strength of the relationship between the quantitative parameters. P-value <0.05 was considered statistically significant. All analysis was done using IBM SPSS Statistics for Windows, Version 24.0 (Released 2016; Armonk, New York, United States).

## Results

**Figure 2:**
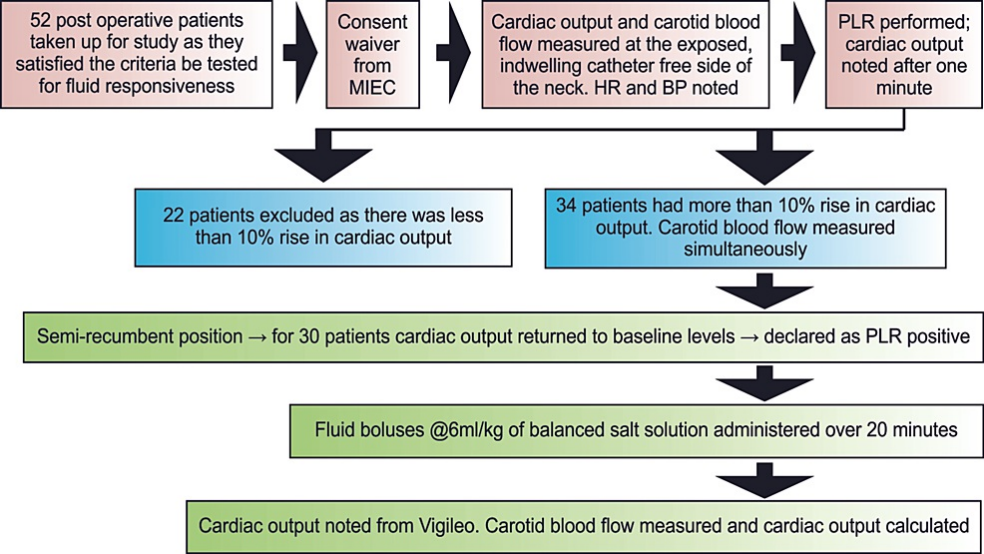
Flow diagram showing the study process MIEC: Medanta Institutional Ethics Committee; PLR: passive leg raise; HR: heart rate; BP: blood pressure

Out of the 52 patients who underwent elective major abdominal surgery and were admitted to the ICU on mechanical ventilator support, 30 patients fulfilled the inclusion criteria and were enrolled for analysis (Figure 2).

Out of the 30 patients analyzed in the study, 25 (83.3%) were male and five (16.7%) were female. Most patients were in the age groups of 41-50 years and 51-60 years, mean (± SD) age being 52.93 (±8.13) years. Sixteen patients (53.3%) were on vasopressors and 14 patients (46.7%) were not (Table 1).

**Table 1:** Demographic characteristics

Parameters	Category	Number of patients	Percentage
Age group (years)	≤40	2	6.7
	41-50	11	36.7
	51-60	10	33.7
	>60	7	23.3
Gender	Male	25	83.3
	Female	5	16.7
BMI (kg/m^2^)	18.5-24.9	13	43.3
	25-29.9	17	56.7
Vasopressors	Yes	16	53.3
	No	14	46.7

The increase in mean SBPs was statistically significant post PLR and post fluid administration (Table 2). 

**Table 2:** Comparison of heart rate, systolic and diastolic blood pressure before and after PLR after fluid bolus PLR: passive leg raise

Hemodynamic variables	Pre-PLR	Post-PLR	Post fluid bolus	P-value (Pre-PLR vs. Post-PLR)	P-value (Pre-PLR vs. Post-fluid)
Heart rate (bpm)	95.53±23.73	97.6±19.17	93.93±19.18	0.386	0.545
Systolic blood pressure (mmHg)	112.2±15.57	118.7±14.96	121.93±13.96	0.001	<0.001
Diastolic blood pressure (mmHg)	60±10.37	61.9±10.13	62.53±8.07	0.242	0.232

**Figure 3:**
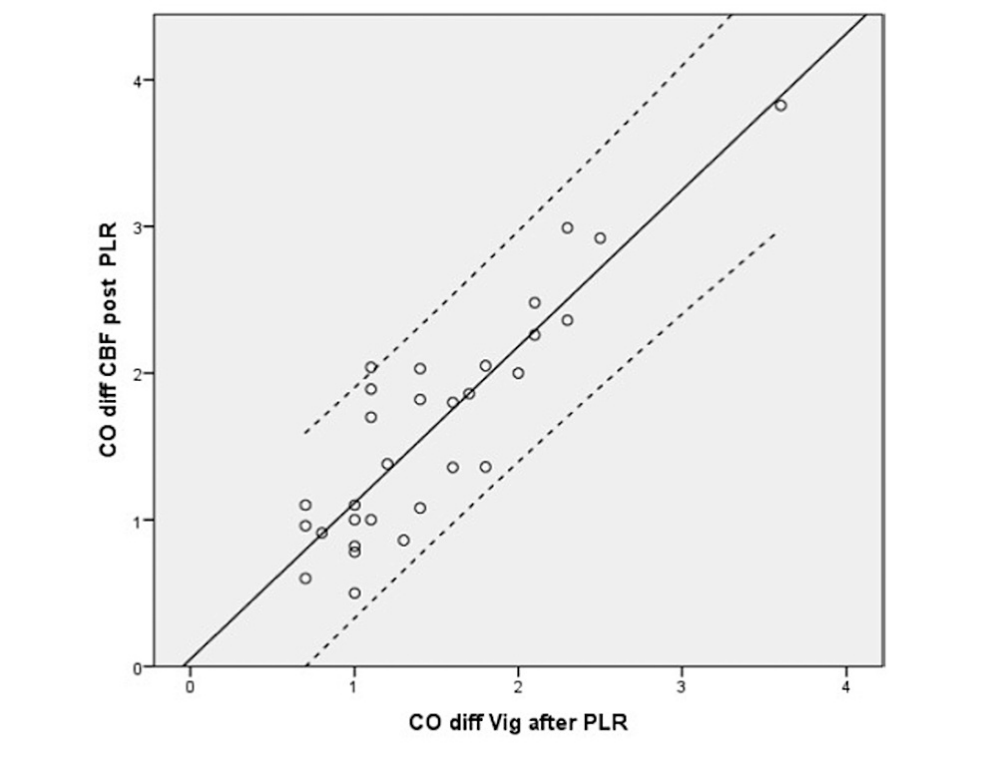
Correlation of change in cardiac output before and after PLR from carotid blood flow versus Vigileo/FloTrac®* r= 0.884 r: correlation coefficient; CO: cardiac output; diff: change; CBF: carotid blood flow; PLR: passive leg raise; Vig: Vigileo/Flotrac *Vigileo monitor/FloTrac® sensor (Edwards Lifesciences, Irvine, California, United States)

**Figure 4:**
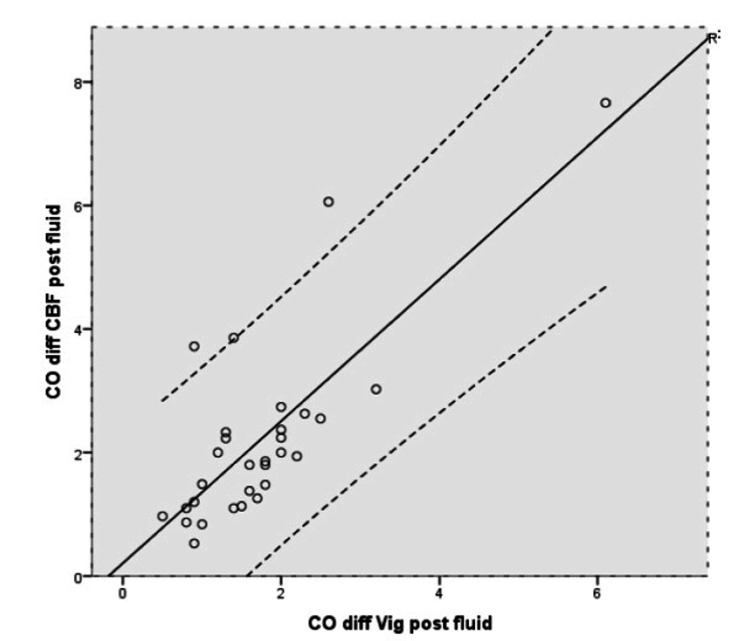
Correlation of change in cardiac output after fluid expansion, from CBF versus Vigileo/FloTrac®* r= 0.781 r: correlation coefficient; CO: cardiac output; diff: change; CBF: carotid blood flow; Vig: Vigileo/Flotrac *Vigileo monitor/FloTrac® sensor (Edwards Lifesciences, Irvine, California, United States)

The change (increase) in CO post PLR and post-fluid bolus administration from the baseline (pre-PLR) was statistically significant and the measurements derived correlated positively between the two groups, that is the arterial waveform-based technique (Vigileo) and the CBF-based derivation. The correlation was stronger in the group without the vasopressors versus group of patients with vasopressors (Tables 3, 4) (Figures 3, 4).

**Table 3:** Cardiac output before and after PLR, and after fluid bolus PLR: passive leg raise

Cardiac output (l/min)	Pre-PLR	Post-PLR	Post fluid bolus	P-value (Pre-PLR vs. Post-PLR)	P-value (Pre-PLR vs. Post-fluid)
Vigileo	7.657±1.445	9.137±1.76	9.393±1.7685	<0.001	<0.001
Carotid blood flow	8.101±1.656	9.72±1.99	10.306±2.256	<0.001	<0.001

**Table 4:** Correlation of change in cardiac output after PLR and after fluid bolus when compared to before PLR * Correlation is significant at the 0.05 level (2-tailed); ** Correlation is significant at the 0.01 level (2-tailed) PLR: passive leg raise

	Correlation Coefficient		
	Over-all	Without vasopressors	With vasopressors
Cardiac output change before and after PLR: Vigileo versus carotid blood flow	0.884**	0.913**	0.834**
Cardiac output change before PLR and after fluid: Vigileo versus carotid blood flow	0.781**	0.544*	0.533*

**Figure 5:**
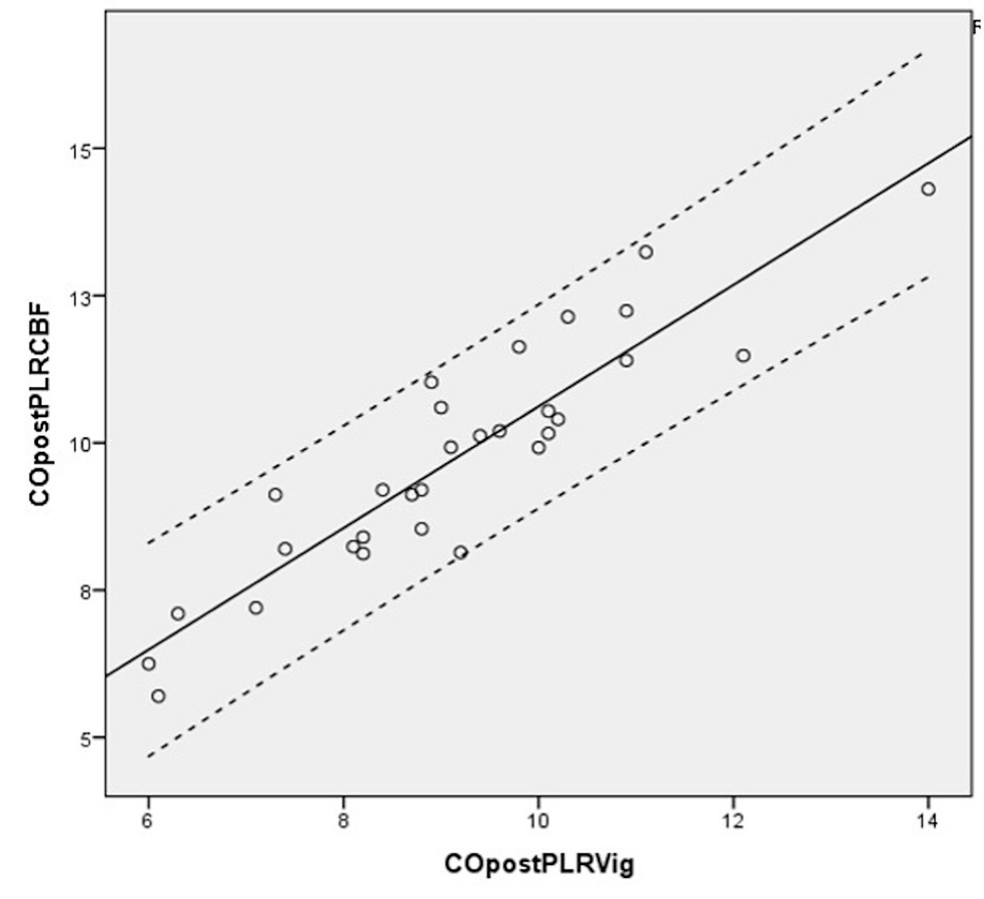
Correlation between mean CO derived from Vigileo* versus CBF post PLR r=0.913 r: correlation coefficient; CO: cardiac output; CBF: carotid blood flow; PLR: passive leg raise; Vig: Vigileo/Flotrac *Vigileo monitor/FloTrac® sensor (Edwards Lifesciences, Irvine, California, United States)

**Figure 6:**
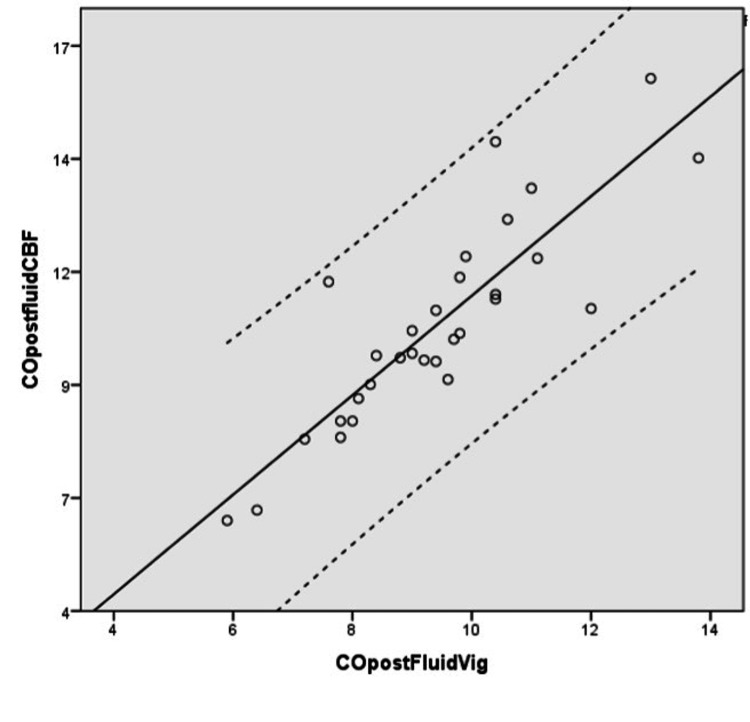
Correlation between mean CO derived from Vigileo* versus CBF post fluid bolus r= 0.863 r: correlation coefficient; CO: cardiac output; CBF: carotid blood flow; PLR: passive leg raise; Vig: Vigileo/Flotrac *Vigileo monitor/FloTrac® sensor (Edwards Lifesciences, Irvine, California, United States)

There was significant correlation between mean CO measurements derived from two techniques, before PLR, after PLR and after fluid expansion (Table 5) (Figures 5, 6).

**Table 5:** Correlation pre-PLR, post-PLR, post-fluid expansion between Vigileo* and CBF-derived CO CO: cardiac output; CBF: carotid blood flow; PLR: passive leg raise *Vigileo monitor/FloTrac® sensor (Edwards Lifesciences, Irvine, California, United States)

CO Correlation between		Correlation coefficient	Significance
Pre-PLR Vigileo	Pre-PLR Carotid Blood Flow	0.881	< 0.001
Post-PLR Vigileo	Post-PLR Carotid Blood Flow	0.913	< 0.001
Post-fluid Vigileo	Post-fluid Carotid Blood Flow	0.863	< 0.001

 

## Discussion

Minimally invasive or non-invasive methods are increasingly being used at the patient's bedside for assessment of fluid responsiveness and avoiding the risks associated with the invasive methods. To avoid excess fluid administration, a positive PLR test has been used to indicate fluid responsiveness. Carotid artery Doppler is a non-invasive, reproducible bedside monitor, that can be utilized as a tool to assess fluid responsiveness by evaluating changes in carotid flow time and CBF during inspiration and expiration.

In our study conducted on 30 post-operative patients as per inclusion criteria, most of them were males and belonged to the age group of 40-60 years. All of them were on ventilator support and 16 were on vasopressor support at the start of recruitment. 

We found that the change in CO from FloTrac/Vigileo correlated positively with the change in CO measured from CBF before and after PLR and also after fluid bolus administration with a stronger correlation in the group without vasopressor support. Also, there was a significant correlation between CO measurements derived from the two techniques before PLR, after PLR, and after fluid bolus, which was our secondary objective. 

Previous studies have found a variable correlation of CO derived from CBF versus invasive techniques. Ma et al. have found a moderate correlation of change in CO derived from CBF in patients who underwent right heart catheterization [20].

In a group of 34 critically ill patients, Marik et al. measured carotid and brachial blood flows to assess fluid responsiveness induced by PLR and observed that CBF increased by 79% and brachial blood flow by 12% in patients who had an increment in stroke volume index of 29.8% after PLR [19]. They also noted that in hemodynamically unstable volume responders, there was a preferential distribution of blood toward the carotid circulation (and brain) and away from the peripheries (brachial circulation) following the fluid bolus.

Gassner and fellow researchers studied 36 patients admitted to the surgical and cardiothoracic ICU and found a good correlation of CO between carotid Doppler-based techniques and pulmonary artery catheter readings [21]. There was also a good correlation between carotid artery-based CO with pulse contour analysis.

Peng et al. studied 148 patients admitted to the ICU with mixed diagnoses, which included postoperative, septic shock, multiple trauma, and respiratory failure [22]. They did not find significant differences in CO measurements derived from TTE and CBF and concluded that CBF may be used to obtain CO when TTE yields a poor image. However, the correlation between carotid CO and TTE was weak in patients with septic shock, multiple trauma, and respiratory failure.

The findings of the current study are in line with the findings of Marik et al. [19], Ma et al. [20], Peng et al. [22], and Gassner et al [21]. Gassner et al. had postoperative surgical patients from cardiothoracic and surgical ICUs [21]; the current study cohort also had patients with elective major abdominal surgery. Marik et al. studied the CBF in septic patients and compared it with bioreactance-derived cardiac index [19]. While Ma et al. studied healthy volunteers in a catheterization laboratory [20], Peng et al. compared echocardiography-based cardiac output in ICU patients with mixed diagnoses [22]. Since septic shock has a mixed etiology of shock, the correlation between invasive cardiac output and carotid Doppler-based findings might be mixed. However, in the studies that have taken postoperative patients, there was a good correlation between carotid Doppler-based CO and invasive CO. The results of the present study are different from other ones mainly because our patients had similar postoperative status and all of them were on ventilators. 

The limitation of the current study is that it was a single-centre study and considered only elective post-operative patients.

## Conclusions

A significant positive correlation of the absolute mean CO as well as the change in CO pre- and post-interventions (that is, PLR and fluid bolus administration) was found, as measured by pulse contour analysis (Vigileo) and by CBF in post-surgical patients. Pulse wave Doppler of CBF could be used as a surrogate for invasive measures of CO measurement for the prediction of fluid responsiveness in this subgroup. Larger scale, multi-center studies on different subgroups of patients in terms of their illness are needed to evaluate this further and validate our findings.
